# Neonatal Respiratory Distress Secondary to Meconium Aspiration Syndrome

**DOI:** 10.3390/children8030246

**Published:** 2021-03-23

**Authors:** Arielle L. Olicker, Thomas M. Raffay, Rita M. Ryan

**Affiliations:** Department of Pediatrics, Case Western Reserve University, UH Rainbow Babies & Children’s Hospital, 11100 Euclid Ave, Cleveland, OH 44106, USA; Thomas.Raffay@UHhospitals.org (T.M.R.); rita.ryan@uhhospitals.org (R.M.R.)

**Keywords:** meconium aspiration syndrome, respiratory distress, surfactant

## Abstract

Infants born through meconium-stained amniotic fluid (MSAF) are 100 times more likely than infants born through clear amniotic fluid to develop respiratory distress in the neonatal period. Meconium aspiration syndrome (MAS) is a common cause of respiratory distress in term and post-mature neonates. MAS is defined as respiratory distress accompanied by a supplemental oxygen requirement in an infant born with MSAF, in the absence of any other identified etiology to explain the symptoms. Therapy for MAS is supportive, and should be tailored to each infant’s specific pathophysiology. In cases of MAS with severe persistent pulmonary hypertension of the newborn (PPHN), patients may remain hypoxic despite aggressive ventilation, and in these cases surfactant, inhaled nitric oxide (iNO) and extracorporeal membrane oxygenation (ECMO) can be life-saving. Long-term prognosis for MAS is more related to severity of initial hypoxemia and possible neurological insult than to the pulmonary pathology.

## 1. Introduction

Meconium aspiration syndrome (MAS) is a common cause of neonatal respiratory distress in term and post-mature neonates.

Meconium is the stool that forms in the developing intestines throughout fetal life. It is thick and viscous, composed of desquamated intestinal epithelial cells and cellular debris, minerals, lanugo, salivary, gastric, pancreatic and intestinal secretions, mucous, bile and bile acids, fetal vernix, blood, enzymes including α1-antitrypsin and phospholipase A2, and amniotic fluid [1,2]. Meconium staining of amniotic fluid occurs in approximately 10–13% of normal pregnancies, and approximately 4% of these infants subsequently develop respiratory distress [1,3]. Infants born through meconium-stained amniotic fluid (MSAF) are 100 times more likely than infants born through clear amniotic fluid to develop respiratory distress in the neonatal period, even in the absence of antenatal fetal heart rate abnormalities or the need for neonatal resuscitation [1,4,5,6]. While some babies who pass meconium in utero show no signs of distress or depression at the time of delivery, MSAF is generally thought of as a sign of fetal distress, and is associated with other conditions related to fetal distress: placental insufficiency, cord compression, preeclampsia, oligohydramnios, small for gestational age (SGA), and maternal substance abuse (especially tobacco and cocaine). Evidence suggests that during times of umbilical cord compression, there is increased parasympathetic activity causing increased vagal tone, which triggers intestinal peristalsis and relaxation of the anal sphincter, ultimately resulting in meconium passage [1,7]. The incidence of MAS has decreased over time, which is largely thought to be secondary to evolving obstetric practices in developed countries, preventing pregnancies from continuing beyond 41 weeks [4,5,6]. When meconium staining of amniotic fluid is accompanied by a non-reassuring fetal heartrate tracing, there is a much greater likelihood of associated neonatal hypoxia, and associated morbidity. However if fetal heartrate tracing remains normal, and meconium passage is not accompanied by acidosis, outcomes are generally favorable [1].

### 1.1. Etiology

Fetal intestinal contents are frequently freely passed into amniotic fluid early in gestation, before the innervation of the GI tract fully migrates distally to the anal sphincter. This stops at approximately 20 weeks of gestation, and passage of meconium into amniotic fluid is rare between 20 and 34 weeks gestation [8]. The incidence of meconium staining becomes much higher with increasing fetal maturity, affecting only 2% of infants born < 37 weeks’ gestation, but as many as 44% of born > 42 weeks gestation [1,8].

Lung fluid predominantly moves in an outward direction during fetal breathing motion, moving from the airways into the oropharynx. During times of fetal distress or asphyxia, the fetus may gasp, causing aspiration of amniotic fluid, and if the distress is accompanied by meconium passage, particulate matter may be aspirated into the trachea and bronchi. Following delivery, when air breathing begins, meconium may migrate from the large airways more distally into the small airways and alveoli. When meconium is aspirated in utero the chest radiograph looks more uniform; if aspirated after birth it often looks patchy [1].

MAS complicates 4% of infants born through MSAF. “Thick” meconium staining, abnormalities in fetal heart rate tracing, low Apgar score at 5 min of life, instrument assisted delivery, emergency caesarian section and planned home birth are all factors that increase the risk of the development of MAS [1,6,9]. Because meconium passage is much more common at term and beyond, MAS tends to be a disease of term and post-term neonates. MAS is defined by the presence of four clinical criteria in the setting of MSAF [2].

1. Respiratory distress (tachypnea, grunting and/or retractions);
2. Requirement for supplemental oxygen to maintain hemoglobin oxygen saturation >92%;
3. Requirement for supplemental oxygen beginning prior to 2 h of life, and lasting at least 12 h;
4. Absence of congenital anomalies of the airway or heart.

Severity of MAS is classified as mild, moderate or severe. Mild MAS as defined as requiring < 40% oxygen for <48 h. MAS is moderate if the infant requires > 40% oxygen and/or supplemental oxygen >48 h but is not complicated by pulmonary air leaks. MAS is severe if an infant requires mechanical ventilation for more than 48 h [10].

### 1.2. Pathophysiology

**Figure 1:**
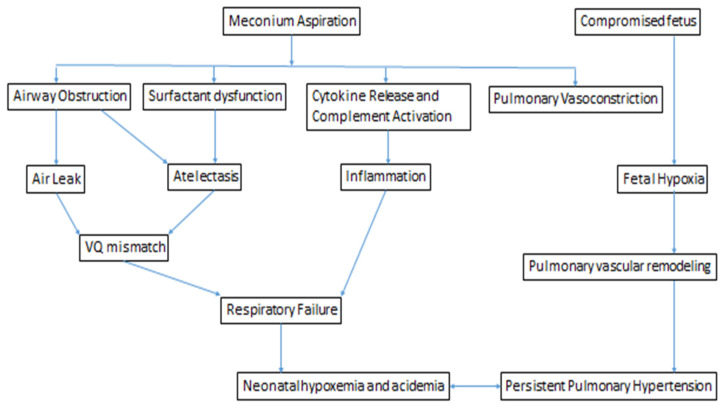
Pathophysiology of persistent pulmonary hypertension of the newborn as a result of fetal compromise and meconium aspiration.

When meconium is aspirated, it has the potential to affect the lungs through three mechanisms: airway obstruction, inflammation, and surfactant dysfunction [1,11]. During the first 15 min following delivery, meconium primarily obstructs the large airways. This leads to increased airway resistance, decreased pulmonary compliance, acute hypoxemia, hypercapnia, and respiratory acidosis. After approximately 60 min, meconium migrates distally to the terminal bronchioles and alveoli, causing atelectasis, inflammation, activation of the complement cascade, cytokine production, and inactivation of pulmonary surfactant [1] (Figure 1).

### 1.3. Airway Obstruction

**Figure 2:**
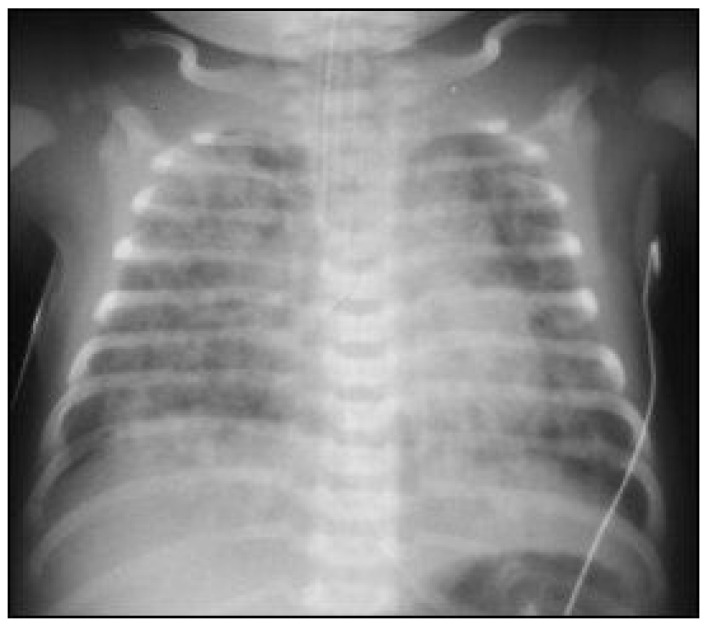
Chest radiograph demonstrating diffuse patchy opacification of the lungs, an example from an infant with meconium aspiration.

When meconium is aspirated into the trachea and bronchi, the more distal airways and alveoli initially remain open. If the obstruction is complete, the airways and alveoli distal to the obstruction collapse, causing post-obstructive atelectasis and V/Q mismatch. However, if the airways are only partially obstructed, negative intrathoracic pressures during inspiration may allow air to pass beyond the obstruction to the distal airspaces. During passive expiration with intrathoracic pressure returning to baseline, the airway may become completely obstructed by meconium, creating a “ball-valve” air-trapping effect, hyperinflation, and may lead to pulmonary air leaks (pneumothorax and/or pneumomediastinum), which are seen in as many as 10–30% of infants with MAS. Commonly, both conditions are present in a patchy pattern, with areas of hyperinflation surrounded by areas of atelectasis [1,12,13]. On chest x-ray, this is classically described as a “salt and pepper” appearance (Figure 2).

### 1.4. Inflammation

While meconium is made up entirely of components derived from the host, gastrointestinal tract contents are considered to be extra-corporeal by the immune system, and are not recognized as “self” [2]. Within hours of meconium aspiration, neutrophils and macrophages are present within the airways and alveoli [1,14]. Multiple components of meconium are toxic to lung tissue, and induce a potent inflammatory response causing a chemical pneumonitis, and triggering a systemic inflammatory response that parallels that of neonatal sepsis. Heme, free fatty acids, bile, bilirubin, and cholesterol all have the potential to induce pulmonary inflammation directly by increasing chemotaxis of neutrophils, and inducing cytokine release from alveolar cells [1,2]. MSAF contains high levels of tumor necrosis factor (TNF), interleukin-1 β (IL-1β), IL-6, and IL-8 which are involved in chemotactic activity of leukocytes [1,14]. Phospholipase A2 releases arachidonic acid from phospholipids in cell membranes, and can directly damage alveolar cells [2]. Meconium aspiration also triggers creation of reactive oxygen and nitrogen species, and activation of the complement cascade. In addition, the hypoxia related to meconium aspiration, and ventilator associated barotrauma as a result of the support required, may also contribute to the inflammatory response seen in MAS [1,2].

### 1.5. Surfactant Dysfunction

Functional surfactant deficiency can occur in MAS due to decreased surfactant production and disruption of surfactant function by fatty acids present in meconium, and this is likely partially responsible for the alveolar collapse seen in MAS [1,15]. Oxidative stress and inflammation allow for leak of plasma proteins across the capillary alveolar membrane, destroying type II pneumocytes responsible for surfactant production. Cholesterol, bile salts, bilirubin, free fatty acids, and proteolytic enzymes may all contribute to altered function of surfactant produced prior to the introduction of meconium to the lungs [1,16]. Meconium alters the structure of the major phospholipid component of surfactant, dipalmitoylphosphatidylcholine (DPPC), causes fragmentation of the phospholipid bilayer, and disrupts function of the liposomes. Meconium also increases minimum surface tension of binary surfactant lipid monolayers: DPPC combined with palmitoyloleoylphosphatidylcholine, phosphatidylethanolamine, or phosphatidylglycerol [9]. Surfactant deficiency results in increased alveolar surface tension, decreased pulmonary compliance, atelectasis, V/Q mismatch, and ultimately hypoxia [1,16,17].

### 1.6. Persistent Pulmonary Hypertension of the Newborn

MAS is often complicated by pulmonary vasoconstriction and severe pulmonary hypertension, which is a significant contributor to morbidity and mortality [11,13]. The exact mechanism by which this occurs is controversial, but it has been speculated that it is precipitated, at least in part, by fetal and neonatal hypoxia leading to pulmonary vascular constriction and eventually remodeling. In addition, animal models suggest that when the lung parenchyma is exposed to meconium pulmonary vasoconstrictor hormonal factors, such as thromboxane A2, angiotensin II and cytokines are released, which in turn cause acute pulmonary vasoconstriction and pulmonary hypertension [1].

### 1.7. Clinical

Infants who develop MAS often have clinical signs of post-maturity (evidence of weight loss, cracked or peeling skin, long nails), SGA, and heavy yellow staining of the skin, nails, and umbilical cord. Hypoxemia that precipitates passage of meconium in utero can also result in neurological and respiratory depression, and perinatal depression is often the predominant clinical finding at the time of delivery; these infants may require therapeutic hypothermia for neuroprotection. Respiratory distress is always present and may be severe, exhibiting tachypnea, cyanosis, grunting, alar flaring, and/or intercostal retractions. Infants may have the appearance of a barrel chest upon visual inspection, and rales may be present upon auscultation [1]. Infants born through MSAF are more likely to suffer from any respiratory illness of infancy than are infants born with clear amniotic fluid, and Wiswell et al. postulates that, rather than being separate entities, these other respiratory illnesses of infancy may actually be a spectrum of MAS [18]. Further, the risk factors for MAS overlap quite a bit with the risk factors for these other respiratory illnesses, such as pneumonia, purulent fluid aspiration, blood aspiration, pneumothorax, and pneumomediastinum, and such neonatal respiratory illnesses may clinically mimic one another [1,10,18].

Arterial blood gases of infants with MAS will reveal hypoxemia and may reveal signs of right-to-left shunting across the PDA due to increased pulmonary vascular tone. This may be initially accompanied by respiratory alkalosis due to tachypnea and hyperventilation, but over time infants often develop respiratory acidosis due to respiratory failure. Hypoxemia over time causes the infant to have a concurrent metabolic acidosis due to anaerobic metabolism of the tissues. Hypoxemia and acidemia each cause pulmonary vascular constriction and decreased pulmonary blood flow, and decreased pulmonary blood flow in turn contributes to increasing hypoxemia and metabolic acidosis. This incites a spiral effect of pulmonary hypertension in MAS. Severe pulmonary hypertension is often present in MAS, and is a significant contributor to morbidity [1].

## 2. Management

### 2.1. Delivery Room

Fetuses at >41 weeks gestational age with non-reassuring fetal heart rate tracings and MSAF require close antenatal monitoring. Amnioinfusion, an obstetric procedure through which normal saline or lactated ringers is instilled into the uterus to replace amniotic fluid, has been studied in the setting of oligohydramnios with amniotic fluid that has been thickly stained by meconium. The intention is for amnioinfusion to prevent MAS by diluting the meconium and making the consistency thinner, and by allowing for relief of cord compression due to oligohydramnios [1]. In a meta-analysis by Pierce et al. of studies evaluating amnioinfusion for prevention of meconium MAS in cases of moderate or thick meconium staining, there were two distinct outcome patterns seen. When at-risk infants are delivered with the availability of standard peripartum care and expert neonatal care, amnioinfusion did not significantly improve outcomes. However, in resource-poor settings with limited availability of antenatal and neonatal care, amnioinfusion significantly decreased the rates of MAS, the need for caesarian section, the presence of meconium below the vocal cords, neonatal acidemia, and did not increase the rate of chorioamnionitis [19]. A large multicenter randomized controlled trial by Fraser et al., published in 2005, concluded that amnioinfusion in the presence of MSAF did not reduce the risk of perinatal death, or moderate or severe MAS [20]. Currently, ACOG does not recommend routine use of prophylactic amnioinfusion in the presence of meconium-stained amniotic fluid for the prevention of meconium aspiration syndrome [21].

It was previously routine practice to suction infants’ oro- and nasopharynx at the perineum following delivery of the head but prior to delivery of the shoulders; however, studies have shown that this practice does not decrease the incidence of MAS, even in the highest risk infants, and may contribute to other morbidities such as desaturation, bradycardia, and pneumothorax [1,22]. Therefore, this practice is not endorsed by the International Consensus on Cardiopulmonary Resuscitation, and has been recommended against as of the 5th edition of the Neonatal Resuscitation Program (NRP) in 2006 [1,11,23].

Recommendations by the NRP for the management of infants born through MSAF have evolved over time and, with the availability of new observational and clinical trial data, recommendations are revised approximately every 5 years [24]. Historically, NRP has recommended endotracheal intubation and suctioning with a meconium aspirator in all infants born through MSAF for prevention of MAS. The 5th edition of NRP changed this recommendation to only routinely intubate for tracheal suctioning for infants who are born non-vigorous [25]. This was updated again with the most recent, 7th edition, of NRP, which cites that there is insufficient evidence to recommend routine endotracheal intubation and suction in even non-vigorous infants born through MSAF for the prevention of MAS [23]. Thus the current recommendation is to initiate routine NRP, with an emphasis on initiating positive pressure ventilation (PPV) during the first minute of life when appropriate. However, the 7th edition of NRP does note that intubation for tracheal suctioning may be necessary if PPV does not allow for adequate ventilation, and it is suspected that this is due to mechanical obstruction with meconium [11,23]. It is anticipated that the 8th edition of NRP will be released in 2021, and will again address the delivery room management of infants born through MSAF.

### 2.2. NICU

The mainstays of therapy for MAS are supportive care, particularly for the respiratory and cardiovascular systems, while the immune system works to clear the meconium and heal the lungs. In order to prevent complications of MAS, it is critical that oxygenation, and systemic blood pressure and perfusion are adequately supported.

Atelectasis, pneumonitis, air trapping, and air leaks all cause ventilation–perfusion (V/Q) mismatch, and subsequently hypoxia, hypercapnia, and acidosis. Chronic hypoxemia in utero may lead to pulmonary vascular remodeling, and this combined with the hypoxia and hypercapnia of meconium aspiration predispose these infants to persistent pulmonary hypertension of the newborn (PPHN) [15].

There is no evidence-based, universally agreed upon, ventilation strategy specific to MAS; rather, an individual infant’s respiratory support should be based on individual clinical presentation. Frequent arterial blood gases are necessary to closely monitor and guide respiratory support needs to maintain pH, PaO_2_ and PaCO_2_ all within targeted ranges. In infants with MAS and significant hypoxemia without signs of PPHN, the predominant pathophysiology is likely V/Q mismatch due to intrapulmonary shunting of blood to poorly ventilated areas of the lung. In infants with MAS without PPHN, the goal of mechanical ventilation is to achieve adequate oxygenation and CO_2_ elimination as gently as possible, to avoid gas trapping, hyperinflation, and pulmonary air leaks. The goals in this case are to maintain pH 7.30–7.40, PaO_2_ 60–80 and PaCO_2_ 40–50. If PPHN is present, it is important to avoid hypoxemia, aiming for PaO_2_ 60–80 mmHg, although animal data suggest levels of PaO_2_ > 60 do not further lower pulmonary vascular resistance [26]. In MAS with PPHN cases, inhaled nitric oxide (iNO) may improve pulmonary blood flow, and improve oxygenation. Because of the risk of gas trapping and air leaks, it is preferable to increase FiO_2_ before increasing positive end expiratory pressure (PEEP) or peak inspiratory pressure (PIP) [15]. If oxygenation and ventilation are refractory to conventional mechanical ventilation, or an air leak is present, either high frequency oscillatory ventilation (HFOV) or high frequency jet ventilation (HFJV) may be more effective [1,15]. There is also a suggestion that HFJV may improve meconium clearance [15].

Infants with severe MAS may benefit from exogenous surfactant administration. There is evidence to support animal-derived surfactants administered to infants with MAS decrease the need for extra-corporeal membrane oxygenation (ECMO) [27,28] and one study demonstrated decreased length of hospital stay with administration of exogenous surfactant [4,6]. However, MAS treatment with surfactant has not shown a statistical difference in duration of supplemental oxygen, duration of mechanical ventilation, incidence of pneumothorax, pulmonary interstitial emphysema, chronic lung disease, or need for oxygen at discharge [1,6,15]. Dargaville et al. conducted a randomized controlled trial of pulmonary lavage with exogenous surfactant in infants on mechanical ventilation for MAS, and found that while there was no difference in duration of respiratory support between the two groups, there was a reduced risk of death or need for ECMO in infants who received surfactant lavage therapy [29,30] and may especially be of use for reduction in mortality in units without the availability of ECMO [3]. In a large, multicenter, randomized controlled trial evaluating surfactant therapy in term infants with respiratory failure, Lotze et al. demonstrated a 29% reduction in the need for ECMO in infants with MAS who received scheduled surfactant when compared to the control group who were not treated with exogenous surfactant [31]. The ideal type of surfactant (animal derived vs. synthetic) and method of administration (bolus administration vs. bronchoalvelolar lavage) remains unknown. When delivering surfactant, close monitoring of cardiopulmonary stability and oxygen saturation is critical, as surfactant has the potential to exacerbate the obstruction of airways, patency of which are already compromised by meconium [15].

Inhaled nitric oxide is often delivered to infants with severe PPHN as a result of MAS in an attempt to allow for pulmonary vascular relaxation. However, it has been suggested that this therapy may be less effective in this subgroup of infants with PPHN due to the physical barrier of meconium in the alveolus [32]. In infants who remain hypoxemic despite aggressive mechanical ventilation and other therapies, including inhaled nitric oxide, extracorporeal membrane oxygenation may be a life-saving measure to allow time for pulmonary recovery [32]. Infants with intractable PPHN due to MAS are considered to be relatively ideal candidates for ECMO therapy, as they are almost all of the appropriate gestational age and weight, have severe, but almost always reversible, lung disease and pulmonary hypertension, would benefit from the “lung rest” that ECMO provides, and overall, infants with MAS have a favorable prognosis with a survival rate of 94% [1,11,32].

While infection does not necessarily accompany MAS, it is possible that sepsis or pneumonia is the impetus for the passage of meconium in utero. Further, on chest x-ray, MAS may have an appearance that is indistinguishable from neonatal pneumonia. For these reasons, an evaluation for sepsis should be performed in infants with MAS, and empiric antibiotics administered while awaiting results [1]. However, if there are no systemic signs of sepsis or a positive blood culture, there is no indication to give a prolonged course of antibiotics simply for meconium aspiration.

### 2.3. Prognosis

Infants with MAS may develop chronic lung disease, which can be a result of either the meconium itself or ventilator associated lung injury. Prior to use of ECMO for MAS, such infants had a higher incidence of asthma later in life [15]. The degree of hypoxia suffered by an infant with MAS predominates the extra-pulmonary prognosis of meconium aspiration syndrome, particularly the neurological outcome [1,15].

## 3. Conclusions

MSAF is considered to be a sign of fetal distress or hypoxia, but only approximately 4% of infants born through MSAF go on to develop MAS. MAS is associated with stigmata of post-maturity, SGA, and other conditions that compromise placental blood flow. Aspirated meconium causes respiratory distress and hypoxemia through the combined effects of obstruction of airways, chemical pneumonitis, and surfactant dysfunction. The 7th edition of NRP recommends initiating NRP with PPV as per the algorithm, only intubating for tracheal suction if unable to provide effective ventilation due to suspected mechanical obstruction. MAS may be associated with severe PPHN. Therapy for MAS is supportive, and should be tailored to each infant’s specific pathophysiology. In cases of MAS with severe PPHN, patients may remain hypoxic despite aggressive ventilation, and in these cases surfactant, inhaled nitric oxide and ECMO can be life saving. Long-term prognosis for MAS is more related to severity of initial hypoxemia and possible neurological insult than to the pulmonary pathology.

## Data Availability

Not applicable.
